# MUSCLE ACTIVATION VARIABILITY AS A DETERMINANT OF GAIT VARIABILITY IN OLDER ADULTS

**DOI:** 10.1093/geroni/igad104.1954

**Published:** 2023-12-21

**Authors:** Dain LaRoche, Purnashree Joshi

**Affiliations:** University of New Hampshire, Durham, New Hampshire, United States; The Plaza Rehabilitation and Nursing Center, Bronx, New York, United States

## Abstract

Gait variability is the step-to-step fluctuations in spatial, temporal, and kinetic gait parameters that reduces the rhythmicity of walking and places older adults at greater risk for falling. Gait variability is influenced by the senses, balance, executive function, strength, and potentially the consistency of neuromuscular activation. The purpose of this study was to test whether older adults with high muscle activation (HMA) variability during walking have greater kinetic, spatial and temporal gait variability than those with low activation variability (LMA). Twenty-seven men and women 65-85 yr walked at normal and maximal speeds on an instrumented treadmill that recorded gait parameters while the electromyogram (EMG) was recorded from vastus lateralis and gastrocnemius lateralis muscles. EMG was recorded over 10 steps and its coefficient of variation was used to identify HMA and LMA groups. Repeated measures ANOVA tested the effects of group (HMA, LMA) and speed (normal, maximal) on gait variability. A significant main effect of speed existed as weight acceptance peak force variability increased 21% (p = 0.014), push off force variability increased 29% (p = 0.015) and step length variability increased 20% (p = 0.02) at maximal speed. A significant interaction existed between speed and group for weight acceptance peak force variability (p = 0.03) as HMA increased 50% while LMA changed little (-4%). Gait variability increased with speed in older men and women, more so for those who exhibit greater muscle activation variability. This suggests the consistency of neuromuscular activation is a factor that affects the rhythmicity of walking.

